# (*Z*)-3-(9-Anthr­yl)-2-(4-nitro-1*H*-imidazol-1-yl)-1-*p*-tolyl­prop-2-en-1-one

**DOI:** 10.1107/S1600536809039221

**Published:** 2009-10-03

**Authors:** Guang-Zhou Wang, Bo Fang, Cheng-He Zhou

**Affiliations:** a Laboratory of Bioorganic and Medicinal Chemistry, School of Chemistry and Chemical Engineering, Southwest University, Chongqing 400715, People’s Republic of China

## Abstract

In the title mol­ecule, C_27_H_19_N_3_O_3_, the imidazole and benzene rings make dihedral angles of 64.72 (4) and 64.02 (4)°, respectively, with the anthracene ring system (r.m.s. deviation = 0.043 Å). The nitro group is coplanar with the imidazole ring [dihedral angle = 1.1 (1)°]. The crystal packing is stabilized by weak π–π inter­actions with centroid–centroid distances of 3.7342 (10) and 3.7627 (9) Å.

## Related literature

For the crystal structures of the chloro and bromo analogues, see: Wang *et al.* (2009 ▶); Lu *et al.* (2009 ▶). For general background to chalcones, see: Vogel *et al.* (2008 ▶). For the synthesis, see: Erhardt *et al.* (1985 ▶).
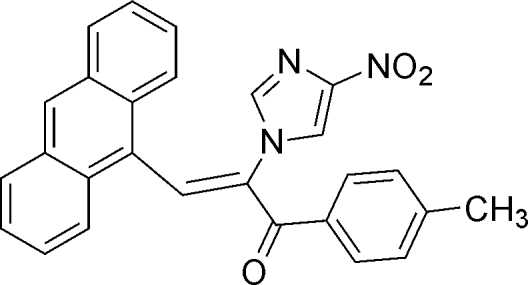


## Experimental

### 

#### Crystal data

C_27_H_19_N_3_O_3_
*M* *_r_* = 433.45
Triclinic, 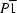

*a* = 7.9335 (9) Å
*b* = 11.2626 (13) Å
*c* = 13.0291 (15) Å
α = 75.454 (2)°
β = 85.763 (2)°
γ = 71.059 (2)°
*V* = 1065.8 (2) Å^3^
*Z* = 2
Mo *K*α radiation
μ = 0.09 mm^−1^
*T* = 298 K
0.36 × 0.23 × 0.10 mm

#### Data collection

Bruker SMART CCD area-detector diffractometer
Absorption correction: multi-scan (*SADABS*; Sheldrick, 1997 ▶) *T*_min_ = 0.968, *T*_max_ = 0.991
12116 measured reflections
4621 independent reflections
3646 reflections with *I* > 2σ(*I*)
*R*_int_ = 0.030

#### Refinement

*R*[*F* ^2^ > 2σ(*F* ^2^)] = 0.044
*wR*(*F* ^2^) = 0.127
*S* = 1.05
4621 reflections
299 parameters
H-atom parameters constrained
Δρ_max_ = 0.18 e Å^−3^
Δρ_min_ = −0.23 e Å^−3^

### 

Data collection: *SMART* (Bruker, 2001 ▶); cell refinement: *SAINT-Plus* (Bruker, 2001 ▶); data reduction: *SAINT-Plus*; program(s) used to solve structure: *SHELXS97* (Sheldrick, 2008 ▶); program(s) used to refine structure: *SHELXL97* (Sheldrick, 2008 ▶); molecular graphics: *SHELXTL* (Sheldrick, 2008 ▶); software used to prepare material for publication: *SHELXTL*.

## Supplementary Material

Crystal structure: contains datablocks I, global. DOI: 10.1107/S1600536809039221/ci2921sup1.cif

Structure factors: contains datablocks I. DOI: 10.1107/S1600536809039221/ci2921Isup2.hkl

Additional supplementary materials:  crystallographic information; 3D view; checkCIF report

## Figures and Tables

**Fig. 1.**
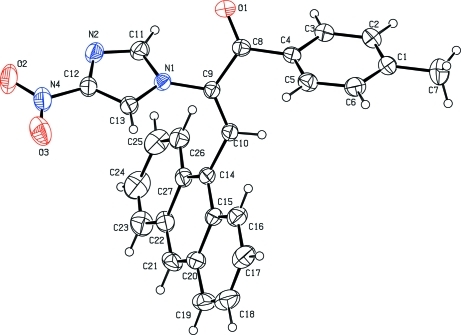
The molecular structure of the title compound, showing the atom-numbering scheme. Displacement ellipsoids are drawn at the 50% probability level.

## supplementary crystallographic information

### Comment

Chalcones or 1,3-diaryl-2-propen-1-ones are natural or synthetic compounds belonging to the flavonoid family (Vogel *et al.*, 2008). They exhibit diverse kinds of biological activities. Hence, chalcones are considered as an important class of therapeutic agents. A series of chalcone derivatives containing a imidazole ring have been synthesized in our lab and crystal structues of some of them have been reported (Lu *et al.*, 2009; Wang *et al.*, 2009). We report here the crystal structure of the title compound.

In the molecular structure of the title compound (Fig. 1), the imidazole and benzene ring of the tolyl group form dihedral angles of 64.72 (4)° and 64.02 (4)°, respectively, with the anthracene ring system (r.m.s. deviation 0.043 Å). The nitro group is coplanar with the imidazole ring [dihedral angle 1.1 (1)°].

The crystal structure is stabilized by weak π-π interactions between imidazole and benzene ring of the tolyl group (centroid to centroid distance = 3.7627 (9) Å) and those between the rings of the anthracene ring system (centroid to centroid distance = 3.7342 (10) Å).

### Experimental

The title compound was synthesized according to the procedure of Erhardt *et al.* (1985). Single crystals suitable for X-ray analysis was grown from a chloroform solution by slow evaporation at room temperature.

### Refinement

H atoms were placed at calculated positions with C-H = 0.93 Å (aromatic) and 0.96 Å (methyl). The *U*_iso_(H) values were set equal to 1.2*U*_eq_(C_aromatic_) and 1.5*U*_eq_(C_methyl_).

### Crystal data

**Table:**

C_27_H_19_N_3_O_3_	*Z* = 2
*M**_r_* = 433.45	*F*(000) = 452
Triclinic, *P*1	*D*_x_ = 1.351 Mg m^−^^3^
Hall symbol: -P 1	Mo *K*α radiation, λ = 0.71073 Å
*a* = 7.9335 (9) Å	Cell parameters from 5053 reflections
*b* = 11.2626 (13) Å	θ = 2.2–28.1°
*c* = 13.0291 (15) Å	µ = 0.09 mm^−^^1^
α = 75.454 (2)°	*T* = 298 K
β = 85.763 (2)°	Plate, yellow
γ = 71.059 (2)°	0.36 × 0.23 × 0.10 mm
*V* = 1065.8 (2) Å^3^	

### Data collection

**Table:**

Bruker SMART CCD area-detector diffractometer	4621 independent reflections
Radiation source: fine-focus sealed tube	3646 reflections with *I* > 2σ(*I*)
graphite	*R*_int_ = 0.030
φ and ω scans	θ_max_ = 27.0°, θ_min_ = 2.2°
Absorption correction: multi-scan (*SADABS*; Sheldrick, 1997)	*h* = −10→10
*T*_min_ = 0.968, *T*_max_ = 0.991	*k* = −14→14
12116 measured reflections	*l* = −16→16

### Refinement

**Table:**

Refinement on *F*^2^	Primary atom site location: structure-invariant direct methods
Least-squares matrix: full	Secondary atom site location: difference Fourier map
*R*[*F*^2^ > 2σ(*F*^2^)] = 0.044	Hydrogen site location: inferred from neighbouring sites
*wR*(*F*^2^) = 0.127	H-atom parameters constrained
*S* = 1.05	*w* = 1/[σ^2^(*F*_o_^2^) + (0.0696*P*)^2^ + 0.061*P*] where *P* = (*F*_o_^2^ + 2*F*_c_^2^)/3
4621 reflections	(Δ/σ)_max_ = 0.012
299 parameters	Δρ_max_ = 0.18 e Å^−^^3^
0 restraints	Δρ_min_ = −0.23 e Å^−^^3^

### Special details

**Table:**

Geometry. All e.s.d.'s (except the e.s.d. in the dihedral angle between two l.s. planes) are estimated using the full covariance matrix. The cell e.s.d.'s are taken into account individually in the estimation of e.s.d.'s in distances, angles and torsion angles; correlations between e.s.d.'s in cell parameters are only used when they are defined by crystal symmetry. An approximate (isotropic) treatment of cell e.s.d.'s is used for estimating e.s.d.'s involving l.s. planes.
Refinement. Refinement of *F*^2^ against ALL reflections. The weighted *R*-factor *wR* and goodness of fit *S* are based on *F*^2^, conventional *R*-factors *R* are based on *F*, with *F* set to zero for negative *F*^2^. The threshold expression of *F*^2^ > σ(*F*^2^) is used only for calculating *R*-factors(gt) *etc*. and is not relevant to the choice of reflections for refinement. *R*-factors based on *F*^2^ are statistically about twice as large as those based on *F*, and *R*- factors based on ALL data will be even larger.

### Fractional atomic coordinates and isotropic or equivalent isotropic displacement parameters (Å^2^)

**Table:**

	*x*	*y*	*z*	*U*_iso_*/*U*_eq_	
C1	0.8022 (2)	0.80103 (14)	0.10371 (11)	0.0625 (4)	
C2	0.9235 (2)	0.68461 (16)	0.09495 (12)	0.0655 (4)	
H2	1.0443	0.6760	0.0895	0.079*	
C3	0.86855 (18)	0.58070 (13)	0.09409 (10)	0.0542 (3)	
H3	0.9521	0.5032	0.0871	0.065*	
C4	0.68960 (16)	0.59098 (11)	0.10360 (9)	0.0443 (3)	
C5	0.56700 (18)	0.70796 (12)	0.11149 (10)	0.0525 (3)	
H5	0.4464	0.7164	0.1176	0.063*	
C6	0.6231 (2)	0.81227 (13)	0.11031 (11)	0.0604 (4)	
H6	0.5394	0.8911	0.1140	0.073*	
C7	0.8646 (3)	0.91345 (19)	0.10604 (18)	0.1030 (7)	
H7A	0.9884	0.8938	0.0878	0.155*	
H7B	0.8486	0.9288	0.1759	0.155*	
H7C	0.7964	0.9893	0.0559	0.155*	
C8	0.63944 (15)	0.47439 (11)	0.10306 (9)	0.0429 (3)	
C9	0.53578 (15)	0.42485 (10)	0.19465 (9)	0.0401 (3)	
C10	0.51789 (15)	0.46279 (11)	0.28466 (9)	0.0424 (3)	
H10	0.5567	0.5329	0.2825	0.051*	
C11	0.55904 (19)	0.21206 (12)	0.15682 (10)	0.0547 (3)	
H11	0.6800	0.1879	0.1413	0.066*	
C12	0.29544 (19)	0.21363 (13)	0.18201 (9)	0.0522 (3)	
C13	0.29627 (17)	0.32641 (12)	0.20011 (9)	0.0479 (3)	
H13	0.2010	0.3902	0.2198	0.057*	
C14	0.44576 (16)	0.41062 (12)	0.38765 (9)	0.0442 (3)	
C15	0.31154 (16)	0.49758 (13)	0.43426 (9)	0.0497 (3)	
C16	0.23060 (19)	0.62857 (14)	0.38169 (12)	0.0604 (4)	
H16	0.2649	0.6593	0.3130	0.072*	
C17	0.1035 (2)	0.71059 (18)	0.42967 (15)	0.0772 (5)	
H17	0.0528	0.7965	0.3936	0.093*	
C18	0.0482 (2)	0.6666 (2)	0.53315 (16)	0.0844 (6)	
H18	−0.0377	0.7238	0.5655	0.101*	
C19	0.1187 (2)	0.5429 (2)	0.58552 (13)	0.0770 (5)	
H19	0.0799	0.5152	0.6538	0.092*	
C20	0.25212 (18)	0.45250 (17)	0.53893 (10)	0.0587 (4)	
C21	0.3253 (2)	0.32471 (18)	0.59118 (10)	0.0668 (4)	
H21	0.2843	0.2958	0.6585	0.080*	
C22	0.4581 (2)	0.23721 (15)	0.54715 (10)	0.0609 (4)	
C23	0.5358 (3)	0.10705 (19)	0.60296 (13)	0.0849 (5)	
H23	0.4934	0.0776	0.6698	0.102*	
C24	0.6697 (3)	0.02531 (18)	0.56107 (15)	0.0961 (6)	
H24	0.7168	−0.0604	0.5983	0.115*	
C25	0.7395 (3)	0.06850 (16)	0.46106 (13)	0.0800 (5)	
H25	0.8346	0.0116	0.4339	0.096*	
C26	0.6691 (2)	0.19203 (13)	0.40416 (11)	0.0588 (4)	
H26	0.7175	0.2190	0.3386	0.071*	
C27	0.52314 (18)	0.28088 (13)	0.44281 (9)	0.0495 (3)	
N1	0.46726 (13)	0.32578 (9)	0.18306 (7)	0.0428 (2)	
N2	0.45809 (17)	0.14128 (11)	0.15594 (9)	0.0602 (3)	
N4	0.1423 (2)	0.17060 (15)	0.18753 (10)	0.0718 (4)	
O1	0.69016 (12)	0.41643 (9)	0.03388 (7)	0.0576 (3)	
O2	0.1606 (2)	0.06711 (14)	0.16772 (11)	0.1051 (5)	
O3	0.00028 (18)	0.24220 (15)	0.21342 (12)	0.0975 (4)	

### Atomic displacement parameters (Å^2^)

**Table:**

	*U*^11^	*U*^22^	*U*^33^	*U*^12^	*U*^13^	*U*^23^
C1	0.0851 (10)	0.0617 (9)	0.0534 (8)	−0.0413 (8)	0.0181 (7)	−0.0175 (7)
C2	0.0631 (9)	0.0805 (10)	0.0669 (9)	−0.0393 (8)	0.0181 (7)	−0.0256 (8)
C3	0.0561 (8)	0.0585 (8)	0.0523 (7)	−0.0224 (6)	0.0110 (6)	−0.0185 (6)
C4	0.0526 (7)	0.0487 (7)	0.0330 (6)	−0.0207 (5)	0.0039 (5)	−0.0070 (5)
C5	0.0549 (7)	0.0518 (7)	0.0487 (7)	−0.0175 (6)	0.0043 (6)	−0.0088 (6)
C6	0.0793 (10)	0.0476 (7)	0.0520 (8)	−0.0192 (7)	0.0090 (7)	−0.0113 (6)
C7	0.1274 (17)	0.0883 (13)	0.1247 (16)	−0.0694 (13)	0.0351 (14)	−0.0441 (12)
C8	0.0430 (6)	0.0459 (6)	0.0378 (6)	−0.0122 (5)	−0.0015 (5)	−0.0083 (5)
C9	0.0436 (6)	0.0390 (6)	0.0385 (6)	−0.0150 (5)	−0.0023 (5)	−0.0078 (5)
C10	0.0473 (6)	0.0432 (6)	0.0399 (6)	−0.0189 (5)	−0.0012 (5)	−0.0094 (5)
C11	0.0663 (8)	0.0466 (7)	0.0539 (7)	−0.0195 (6)	0.0048 (6)	−0.0160 (6)
C12	0.0684 (9)	0.0562 (8)	0.0393 (6)	−0.0335 (7)	−0.0051 (6)	−0.0049 (5)
C13	0.0509 (7)	0.0533 (7)	0.0419 (6)	−0.0211 (6)	−0.0030 (5)	−0.0089 (5)
C14	0.0501 (7)	0.0566 (7)	0.0347 (6)	−0.0276 (6)	−0.0006 (5)	−0.0122 (5)
C15	0.0491 (7)	0.0693 (9)	0.0428 (6)	−0.0290 (6)	0.0004 (5)	−0.0216 (6)
C16	0.0582 (8)	0.0705 (9)	0.0604 (8)	−0.0221 (7)	0.0010 (6)	−0.0276 (7)
C17	0.0632 (9)	0.0857 (11)	0.0916 (12)	−0.0160 (8)	−0.0031 (8)	−0.0463 (10)
C18	0.0562 (9)	0.1257 (17)	0.0902 (13)	−0.0252 (10)	0.0098 (9)	−0.0667 (13)
C19	0.0564 (9)	0.1382 (17)	0.0592 (9)	−0.0450 (10)	0.0129 (7)	−0.0491 (11)
C20	0.0508 (7)	0.0984 (11)	0.0442 (7)	−0.0399 (7)	0.0042 (6)	−0.0274 (7)
C21	0.0708 (9)	0.1096 (13)	0.0367 (7)	−0.0552 (9)	0.0048 (6)	−0.0134 (8)
C22	0.0771 (10)	0.0761 (9)	0.0401 (7)	−0.0446 (8)	−0.0072 (6)	−0.0034 (6)
C23	0.1265 (16)	0.0850 (12)	0.0485 (8)	−0.0569 (12)	−0.0116 (9)	0.0089 (8)
C24	0.151 (2)	0.0634 (10)	0.0639 (11)	−0.0333 (12)	−0.0260 (12)	0.0091 (9)
C25	0.1040 (13)	0.0594 (9)	0.0676 (10)	−0.0148 (9)	−0.0201 (9)	−0.0081 (8)
C26	0.0715 (9)	0.0565 (8)	0.0480 (7)	−0.0221 (7)	−0.0097 (6)	−0.0065 (6)
C27	0.0603 (8)	0.0576 (8)	0.0378 (6)	−0.0308 (6)	−0.0062 (5)	−0.0065 (5)
N1	0.0501 (6)	0.0427 (5)	0.0385 (5)	−0.0179 (4)	−0.0008 (4)	−0.0105 (4)
N2	0.0837 (9)	0.0513 (6)	0.0546 (7)	−0.0321 (6)	0.0017 (6)	−0.0150 (5)
N4	0.0921 (10)	0.0831 (9)	0.0569 (7)	−0.0572 (9)	−0.0091 (7)	−0.0046 (7)
O1	0.0656 (6)	0.0654 (6)	0.0495 (5)	−0.0253 (5)	0.0130 (4)	−0.0253 (5)
O2	0.1427 (12)	0.1039 (10)	0.1091 (10)	−0.0885 (10)	−0.0031 (9)	−0.0309 (8)
O3	0.0747 (8)	0.1167 (11)	0.1149 (11)	−0.0555 (8)	0.0020 (7)	−0.0195 (8)

### Geometric parameters (Å, °)

**Table:**

C1—C2	1.378 (2)	C13—H13	0.93
C1—C6	1.383 (2)	C14—C27	1.4085 (18)
C1—C7	1.509 (2)	C14—C15	1.4099 (17)
C2—C3	1.3779 (19)	C15—C16	1.414 (2)
C2—H2	0.93	C15—C20	1.4327 (18)
C3—C4	1.3852 (18)	C16—C17	1.361 (2)
C3—H3	0.93	C16—H16	0.93
C4—C5	1.3835 (18)	C17—C18	1.405 (3)
C4—C8	1.4923 (16)	C17—H17	0.93
C5—C6	1.3814 (18)	C18—C19	1.339 (3)
C5—H5	0.93	C18—H18	0.93
C6—H6	0.93	C19—C20	1.429 (2)
C7—H7A	0.96	C19—H19	0.93
C7—H7B	0.96	C20—C21	1.377 (2)
C7—H7C	0.96	C21—C22	1.390 (2)
C8—O1	1.2133 (13)	C21—H21	0.93
C8—C9	1.4933 (16)	C22—C23	1.415 (2)
C9—C10	1.3289 (15)	C22—C27	1.4426 (18)
C9—N1	1.4332 (14)	C23—C24	1.344 (3)
C10—C14	1.4772 (15)	C23—H23	0.93
C10—H10	0.93	C24—C25	1.411 (3)
C11—N2	1.3029 (17)	C24—H24	0.93
C11—N1	1.3657 (16)	C25—C26	1.355 (2)
C11—H11	0.93	C25—H25	0.93
C12—C13	1.3510 (17)	C26—C27	1.418 (2)
C12—N2	1.3551 (18)	C26—H26	0.93
C12—N4	1.4381 (18)	N4—O2	1.2174 (17)
C13—N1	1.3563 (15)	N4—O3	1.2346 (18)
			
C2—C1—C6	118.62 (13)	C14—C15—C20	119.28 (13)
C2—C1—C7	120.37 (15)	C16—C15—C20	117.91 (12)
C6—C1—C7	121.01 (15)	C17—C16—C15	121.32 (15)
C1—C2—C3	120.94 (14)	C17—C16—H16	119.3
C1—C2—H2	119.5	C15—C16—H16	119.3
C3—C2—H2	119.5	C16—C17—C18	120.57 (18)
C2—C3—C4	120.40 (13)	C16—C17—H17	119.7
C2—C3—H3	119.8	C18—C17—H17	119.7
C4—C3—H3	119.8	C19—C18—C17	120.34 (16)
C5—C4—C3	118.92 (12)	C19—C18—H18	119.8
C5—C4—C8	123.45 (11)	C17—C18—H18	119.8
C3—C4—C8	117.62 (11)	C18—C19—C20	121.48 (16)
C6—C5—C4	120.24 (13)	C18—C19—H19	119.3
C6—C5—H5	119.9	C20—C19—H19	119.3
C4—C5—H5	119.9	C21—C20—C19	122.36 (14)
C5—C6—C1	120.85 (13)	C21—C20—C15	119.29 (13)
C5—C6—H6	119.6	C19—C20—C15	118.35 (15)
C1—C6—H6	119.6	C20—C21—C22	122.53 (12)
C1—C7—H7A	109.5	C20—C21—H21	118.7
C1—C7—H7B	109.5	C22—C21—H21	118.7
H7A—C7—H7B	109.5	C21—C22—C23	121.93 (14)
C1—C7—H7C	109.5	C21—C22—C27	119.26 (13)
H7A—C7—H7C	109.5	C23—C22—C27	118.78 (16)
H7B—C7—H7C	109.5	C24—C23—C22	121.12 (16)
O1—C8—C4	121.31 (10)	C24—C23—H23	119.4
O1—C8—C9	120.52 (11)	C22—C23—H23	119.4
C4—C8—C9	118.02 (10)	C23—C24—C25	120.55 (16)
C10—C9—N1	121.36 (10)	C23—C24—H24	119.7
C10—C9—C8	122.61 (10)	C25—C24—H24	119.7
N1—C9—C8	115.87 (9)	C26—C25—C24	120.61 (18)
C9—C10—C14	129.89 (10)	C26—C25—H25	119.7
C9—C10—H10	115.1	C24—C25—H25	119.7
C14—C10—H10	115.1	C25—C26—C27	121.12 (14)
N2—C11—N1	112.40 (13)	C25—C26—H26	119.4
N2—C11—H11	123.8	C27—C26—H26	119.4
N1—C11—H11	123.8	C14—C27—C26	123.60 (11)
C13—C12—N2	112.85 (11)	C14—C27—C22	118.59 (13)
C13—C12—N4	125.76 (14)	C26—C27—C22	117.70 (12)
N2—C12—N4	121.39 (13)	C13—N1—C11	106.73 (10)
C12—C13—N1	104.50 (11)	C13—N1—C9	125.53 (10)
C12—C13—H13	127.7	C11—N1—C9	127.71 (11)
N1—C13—H13	127.7	C11—N2—C12	103.51 (11)
C27—C14—C15	121.01 (11)	O2—N4—O3	124.53 (15)
C27—C14—C10	120.56 (11)	O2—N4—C12	118.72 (16)
C15—C14—C10	118.04 (11)	O3—N4—C12	116.74 (13)
C14—C15—C16	122.80 (11)		
			
C6—C1—C2—C3	−0.8 (2)	C14—C15—C20—C19	178.69 (11)
C7—C1—C2—C3	179.16 (15)	C16—C15—C20—C19	−1.83 (17)
C1—C2—C3—C4	−0.9 (2)	C19—C20—C21—C22	−178.42 (12)
C2—C3—C4—C5	1.47 (19)	C15—C20—C21—C22	1.3 (2)
C2—C3—C4—C8	−179.46 (11)	C20—C21—C22—C23	178.14 (14)
C3—C4—C5—C6	−0.28 (18)	C20—C21—C22—C27	0.1 (2)
C8—C4—C5—C6	−179.29 (11)	C21—C22—C23—C24	−176.94 (16)
C4—C5—C6—C1	−1.5 (2)	C27—C22—C23—C24	1.1 (3)
C2—C1—C6—C5	2.0 (2)	C22—C23—C24—C25	1.6 (3)
C7—C1—C6—C5	−177.96 (14)	C23—C24—C25—C26	−2.0 (3)
C5—C4—C8—O1	127.74 (13)	C24—C25—C26—C27	−0.5 (2)
C3—C4—C8—O1	−51.28 (16)	C15—C14—C27—C26	−174.16 (11)
C5—C4—C8—C9	−56.71 (15)	C10—C14—C27—C26	−1.40 (18)
C3—C4—C8—C9	124.27 (12)	C15—C14—C27—C22	2.01 (17)
O1—C8—C9—C10	161.69 (12)	C10—C14—C27—C22	174.76 (11)
C4—C8—C9—C10	−13.90 (17)	C25—C26—C27—C14	179.39 (13)
O1—C8—C9—N1	−13.70 (16)	C25—C26—C27—C22	3.2 (2)
C4—C8—C9—N1	170.72 (10)	C21—C22—C27—C14	−1.75 (18)
N1—C9—C10—C14	5.68 (19)	C23—C22—C27—C14	−179.84 (13)
C8—C9—C10—C14	−169.46 (11)	C21—C22—C27—C26	174.64 (12)
N2—C12—C13—N1	0.92 (14)	C23—C22—C27—C26	−3.45 (19)
N4—C12—C13—N1	−178.45 (11)	C12—C13—N1—C11	−0.61 (13)
C9—C10—C14—C27	59.15 (18)	C12—C13—N1—C9	−178.63 (10)
C9—C10—C14—C15	−127.88 (14)	N2—C11—N1—C13	0.13 (14)
C27—C14—C15—C16	179.91 (11)	N2—C11—N1—C9	178.09 (10)
C10—C14—C15—C16	6.98 (17)	C10—C9—N1—C13	54.97 (16)
C27—C14—C15—C20	−0.64 (17)	C8—C9—N1—C13	−129.58 (11)
C10—C14—C15—C20	−173.57 (10)	C10—C9—N1—C11	−122.63 (14)
C14—C15—C16—C17	−178.85 (12)	C8—C9—N1—C11	52.83 (15)
C20—C15—C16—C17	1.70 (19)	N1—C11—N2—C12	0.41 (14)
C15—C16—C17—C18	−0.4 (2)	C13—C12—N2—C11	−0.83 (14)
C16—C17—C18—C19	−0.7 (2)	N4—C12—N2—C11	178.56 (11)
C17—C18—C19—C20	0.5 (2)	C13—C12—N4—O2	178.97 (13)
C18—C19—C20—C21	−179.54 (14)	N2—C12—N4—O2	−0.34 (19)
C18—C19—C20—C15	0.8 (2)	C13—C12—N4—O3	−1.7 (2)
C14—C15—C20—C21	−1.03 (17)	N2—C12—N4—O3	178.98 (13)
C16—C15—C20—C21	178.45 (12)		
